# Environmental and behavioral modulation of the number of substantia nigra dopamine neurons in adult mice

**DOI:** 10.1002/brb3.163

**Published:** 2013-09-07

**Authors:** Tim D Aumann, Doris Tomas, Malcolm K Horne

**Affiliations:** Florey Institute of Neuroscience and Mental Health, The University of MelbourneParkville, Victoria, Australia, 3010

**Keywords:** Dopamine, midbrain, plasticity

## Abstract

**Background:**

Recent evidence indicates that hypothalamic neurons acquire or lose the capacity to synthesize and release dopamine (DA) in response to environmental stimuli, and this has functional and behavioral consequences for adult rats. We have evidence that neuronal activity, including that driven by afferent input, regulates acquisition and loss of the DA phenotype by substantia nigra pars compacta (SNc) neurons in adult mice.

**Hypotheses** The aims of the present study were to determine whether the environment or behavior regulates the number of SNc DA neurons in adult mice, and whether this is mediated by afferent input.

**Methods:**

Adult mice were subject to two different environments/behaviors: “mating” for 1 week or “environment enrichment” (EE) for 2 weeks; then the numbers of tyrosine hydroxylase (TH, the rate limiting enzyme in DA synthesis) immunopositive (TH+) and immunonegative (TH−) SNc neurons were counted.

**Results:**

More TH+ neurons were present in mated males whereas less TH+ neurons were present in mated females. Also, more TH+ neurons were present in EE males, and this increase was completely abolished by concurrent local infusion of GABA_A_ receptor antagonists.

**Conclusions:**

The number of DA neurons in the adult SNc is not fixed, but readily increases and decreases in response to environmental stimuli and/or behaviors. These changes are mediated by afferent input relaying information about the environment or behavior to SNc neurons.

## Introduction

Behavioral adaptation to our environment is mediated by changes in our nervous system. In adults these changes include altered connectivity between neurons (synaptic plasticity) and limited generation of new neurons (neurogenesis). There is, however, evidence of another form of “plasticity,” which involves neurons changing the levels of expression of, or identity of their neurotransmitter (Zigmond et al. 1980; Baker et al. 1983; Black et al. 1985, 1987; Richard et al. 1988; Biguet et al. 1989; Schalling et al. 1989; Liaw et al. 1992; Aumann et al. 2011; Dulcis et al. 2013). For example, acquisition or loss of the capacity of hypothalamic neurons to synthesize and release dopamine (DA) in response to environmental stimuli has functional and behavioral consequences for adult rats (Dulcis et al. 2013). We too have reported evidence for acquisition and loss of the DA phenotype by adult substantia nigra pars compacta (SNc) neurons in response to altered neuronal activity (Aumann et al. 2008, 2011) or following 6-hydroxy-dopamine (6-OHDA) lesions (Stanic et al. 2003). Our data further suggest that afferent input to SNc regulates this “DA phenotype switching” (Aumann et al. 2011). This implicates behavior and/or the environment in regulating the number of DA neurons in the adult SNc, via afferent input.

The aims of the present study were to determine: (1) whether the number of SNc DA neurons changes in mice undergoing natural behaviors; (2) which kinds of behavior best induce these changes; and (3) whether afferent input is involved.

## Material and Methods

All experimental procedures on animals were approved by the Florey Institute of Neuroscience and Mental Health Animal Ethics Committee and conform to Australia's National Health and Medical Research Council published code of practice for the care and use of animals for scientific purposes (7th edition, 2004).

### Mice

Except where otherwise indicated, all mice in the present experiments were C57BL/6J purchased from the Animal Resources Centre in Western Australia (http://www.arc.wa.gov.au/). They were housed in a temperature-controlled environment with a 12:12 h light:dark cycle and ad libitum access to standard rodent pellets and water. All mice were >8 weeks old (i.e., sexually mature) at the beginning of experimentation and mice in each experiment were age matched and randomly assigned to different experimental groups.

### Mating

Mated mice were housed together in pairs continuously for 7 days in standard mouse boxes. Experimental groups were male–female pair (mated), male–male pair (control males), and female–female pair (control females). No other manipulations were performed except for standard animal husbandry (daily observation and topping up food and water when necessary), which was applied equally across experimental groups. Female mice were checked for pregnancy at the time of killing.

### Environment enrichment

Environment-enriched mice (males only) were housed together in groups of *n* = 6 mice each continuously for 14 days in nonstandard boxes measuring 27 cm wide, 42 cm long, and 16 cm deep. Mice were assigned to one of three different groups: (1) standard housed (SH) comprising litter only; (2) running wheel (RW) comprising SH plus 2 RWs (per cage); and (3) environment enriched (EE) comprising RW plus toys (ropes, ladders, tunnels, and objects) with which to explore, play, climb, hide, and nest. EE mice were also “super-enriched” for 1 h/day 5 days (Monday–Friday)/week. Super enrichment occurred at the same time each day (usually midday during the light cycle) and comprised placement into a larger cage (46 cm wide, 69 cm long, and 40 cm deep) containing novel toys. These toys were cleaned with soapy water and 80% ethanol after each session and a different set of toys was presented at each session. These mice were returned to their EE cage following super enrichment.

### Tissue collection, processing, and immunohistochemistry

Immediately following the behavioral manipulations, mice were killed with sodium pentobarbitone (100 mg/kg i.p.) and perfused intracardially with 37°C heparinized phosphate-buffered saline (PBS) followed by 4% paraformaldehyde in PBS (1.15 mL/g body weight). The brain was removed and placed in PBS containing 30% sucrose for 2–3 days. Serial sections (40 μm thick) were cut through the midbrain and pons in the coronal plane using a cryostat. Every fourth section was incubated in 5% normal goat serum and 0.3% triton X-100 in PBS (30 min), immunoreacted with polyclonal rabbit anti-TH (1:400, Merck Millipore, Billerica, MA, 48 h), polyclonal biotinylated goat anti-rabbit (1:1000, Dako, Glostrup, Denmark, 2 h), avidin-peroxidase (1:500, 1 h), cobalt and nickel intensified diamino-benzidine (0.5 mg/mL, 15 min), then hydrogen peroxide (0.01%, 3–5 min). Sections were mounted on gelatinized microscope slides, air dried, then Nissl stained (neutral red), dehydrated in alcohol, cleared (X-3B), and coverslipped.

### Stereology

The numbers of tyrosine hydroxylase immunopositive (TH+) and tyrosine hydroxylase immunonegative (TH−) cells in the left SNc, ventral tegmental area (VTA), and locus coeruleus (LC) were estimated using unbiased stereological methods (Stereo Investigator, MicroBrightField, VT). TH+ cells were immunoreactive for TH whereas TH− cells were not immunoreactive for TH but were Nissl stained. The stereologist was blind to the treatment received. The SNc, VTA, and LC were identified by the spatial distribution of TH+ cells and anatomical landmarks/boundaries according to the atlas of Paxinos and Watson (Paxinos and Franklin 2004). Counts of TH+ and TH− cells within a counting frame (55 × 55 μm = 3025 μm^2^) were made at regular predetermined intervals (*x* = 140 μm, *y* = 140 μm for SNc; *x* = 100 μm, *y* = 100 μm for VTA and LC) throughout each nucleus in every fourth section. Only those cells with a visible nucleus were counted and glia were excluded on the basis of soma diameter <5 μm.

### Drug infusions

Some mice had an osmotic minipump implanted to infuse a GABA_A_ receptor antagonist (or vehicle) locally into the left SNc and surrounds via a cannula implanted in the midbrain. This surgery was performed immediately prior to the beginning of EE (or SH) and the infusion lasted throughout the 2-week period of EE (or SH). On the day before surgery, ALZET® micro-osmotic pumps (model 1002; DURECT Corporation, Cupertino, CA) attached to ALZET® brain infusion cannulae (brain infusion kit 1) were prefilled with drug or vehicle and immersed in 37°C sterile saline overnight to prime the pumps. Mice were anesthetized with isofluorane (1–1.5%) in air and placed in a stereotaxic headframe. The infusion cannula was introduced through a ∼1.5 mm diameter craniotomy and the cannula tip was placed in the left midbrain at coordinates: 3.0 mm posterior to Bregma, 1.5 mm lateral to the midline, and 4.0 mm below the surface of the brain. The cannula was glued to the skull with dental cement and the attached pump was placed in a subcutaneous “pocket” created in the interscapular region. The location of the tip of the cannula was verified histologically to be in or just dorsal to SNc in each mouse (e.g., asterisks in Fig. 3A).

## Results

### Effects of mating

**Figure 1:**
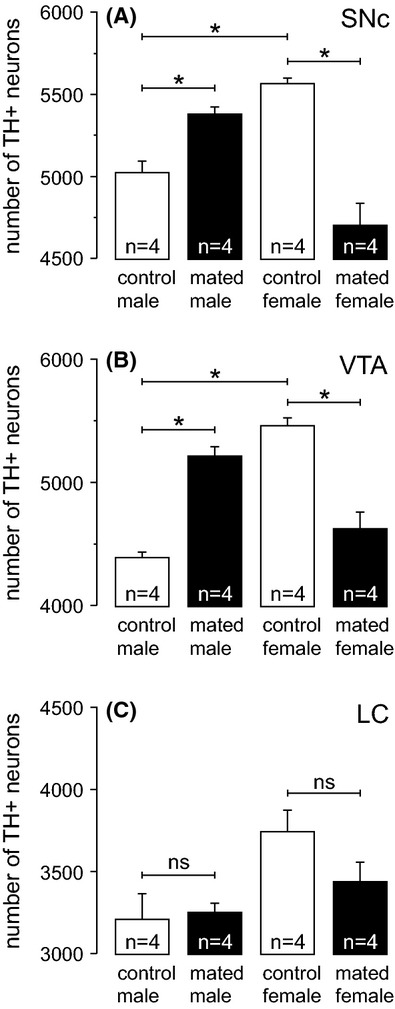
Changes in the number of tyrosine hydroxylase immunopositive (TH+) neurons in the adult mouse midbrain with mating behavior. Mean ± SE number of TH+ neurons in the substantia nigra pars compacta (SNc;A), ventral tegmental area (VTA;B), and locus ceruleus (LC;C). Differences between control and mated mice within each gender were observed only in the midbrain (SNc and VTA), not in the noradrenergic LC. Mated males had more midbrain TH+ neurons whereas mated females had less midbrain TH+ neurons. Also, control females had more midbrain TH+ neurons than control males. *, significant difference, ns, no significant difference (two-way ANOVA with Tukey multiple comparisons, see Table 1). SE, standard error; ANOVA, analysis of variance.

Males paired with females for 7 days (mated males) had significantly more TH+ SNc neurons than males paired with males (control males) (Fig. 1A; Table 1). The magnitude of this increase was ∼8%, or 400 cells. A similar increase was seen in the VTA just medial to SNc (Fig. 1B; Table 1), but TH+ cell numbers were unchanged in another catecholaminergic (but not DAergic) nucleus, the locus ceruleus (LC) (Fig. 1C; Table 1). In contrast, there were approximately 900 fewer TH+ SNc neurons (16%) in females paired with males for 7 days (mated females) than in females paired with females (control females) (Fig. 1A; Table 1). Again the same effect was seen in the VTA (Fig. 1B; Table 1) but not in the LC, where there was no change (Fig. 1C; Table 1). Note also that control females had significantly more TH+ SNc and VTA neurons than control males (Fig. 1A; Table 1).

**Table 1:** Stereological estimates of the number (mean ± SE, *n* = 4/group) of tyrosine hydroxylase immunopositive (TH+) and immunonegative (TH−) neurons in the substantia nigra pars compacta (SNc), ventral tegmental area (VTA), and locus ceruleus (LC) in adult control and mated male and female mice

		TH+	TH−	Total (TH+ and TH−)
SNc	Control male	5021 ± 74	1056 ± 49	6078 ± 57
	Mated male	5378 ± 48*	1146 ± 19	6525 ± 62*
	Control female	5566 ± 36†	959 ± 110	6525 ± 88†
	Mated female	4704 ± 133*	972 ± 142	5676 ± 48*
VTA	Control male	4393 ± 49	585 ± 62	4979 ± 109
	Mated male	5217 ± 80*	992 ± 89*	6208 ± 81*
	Control female	5461 ± 68†	783 ± 52	6245 ± 79†
	Mated female	4628 ± 139*	833 ± 97	5461 ± 161*
LC	Control male	3210 ± 157	1038 ± 81	4248 ± 209
	Mated male	3253 ± 56	879 ± 173	4132 ± 124
	Control female	3742 ± 134	655 ± 142	4397 ± 110
	Mated female	3438 ± 122	936 ± 144	4374 ± 157

There are significant (*P* < 0.001) gender x mating interaction effects on the number of TH+ and Total (TH+ and TH−) neurons in SNc and VTA (two-way ANOVA). There is a significant (*P* < 0.05) main effect of mating and a significant (*P* < 0.05) gender × mating interaction effect on TH− neurons in VTA, but not in SNc. There is a significant (*P* > 0.05) main effect of gender only in LC. SE, standard error; ANOVA, analysis of variance.

* *P* < 0.05, mated versus control;

† *P* < 0.05, control female versus control male (Tukey multiple comparisons).

These mating-induced changes in TH+ neurons were not accompanied by coincident change in the number of TH− SNc neurons in either males or females (Table 1, but see Discussion), apart from an almost doubling of TH− neurons in VTA of mated male mice (Table 1). These findings were reproduced on three different cohorts of mice, including one cohort of Swiss mice (instead of C57BL/6J mice).

### Effects of environment enrichment

**Figure 2:**
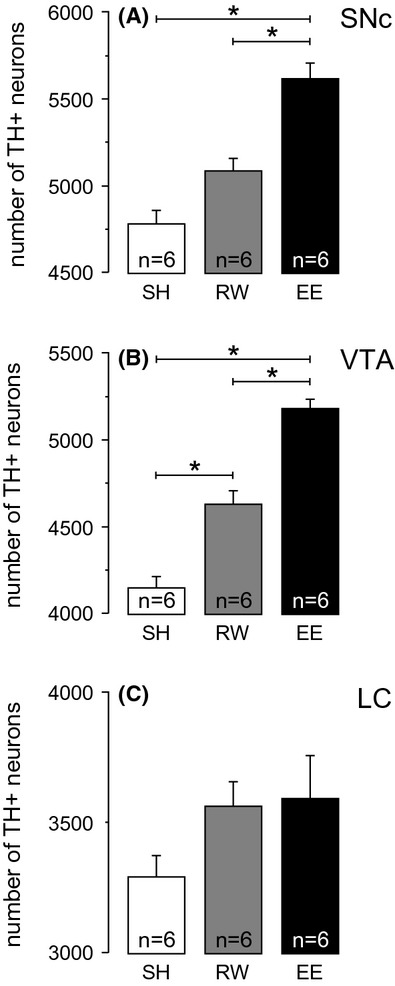
Changes in the number of tyrosine hydroxylase immunopositive (TH+) neurons in the adult mouse midbrain with environment enrichment. Mean ± SE number of TH+ neurons in the substantia nigra pars compacta (SNc;A), ventral tegmental area (VTA; B), and locus ceruleus (LC;C). Data are from adult male mice only. Differences between standard housed (SH), running wheel only (RW), and environment-enriched (EE) mice were observed only in the midbrain (SNc and VTA), not in the noradrenergic LC. More TH+ neurons were observed in RW mice compared with SH mice in VTA, and EE resulted in further increases in TH+ neurons in both SNc and VTA. *Significant difference (one-way ANOVA with Tukey multiple comparisons, see Table 2). SE, standard error; ANOVA, analysis of variance.

Only male mice were subjected to environment enrichment (EE). There was a trend toward an increase in the number of TH+ SNc neurons in running wheel-only (RW) mice compared with SH mice, which lacked statistical significance with this small sample size (Fig. 2A; Table 2). By contrast, the number of TH+ SNc neurons was further increased by the addition of EE to the RW condition, making the difference with both RW and SH significant (Fig. 2A; Table 2). The magnitude of this increase was approximately 500 cells or 10% of the RW population and 850 cells or 17% of the SH population (Fig. 2A; Table 2). Again the same effect was seen in the VTA (Fig. 2B; Table 2) but not in the locus ceruleus (LC), where there was no change (Fig. 2C; Table 2).

**Table 2:** Stereological estimates of the number (mean ± SE, *n* = 6/group) of tyrosine hydroxylase immunopositive (TH+) and immunonegative (TH−) neurons in the substantia nigra pars compacta (SNc), ventral tegmental area (VTA), and locus ceruleus (LC) in standard housed, running wheel, and environment-enriched adult male mice

		TH+	TH−	Total (TH+ and TH−)
SNc	Standard housed	4777 ± 86	1317 ± 100	6095 ± 29
	Running wheel	5088 ± 73	1317 ± 78	6406 ± 26*
	Environment enriched	5615 ± 98*†	1166 ± 62	6782 ± 65*†
VTA	Standard housed	4148 ± 66	855 ± 48	5003 ± 28
	Running wheel	4628 ± 82*	776 ± 94	5404 ± 60*
	Environment enriched	5181 ± 54*†	992 ± 61	6173 ± 90*†
LC	Standard housed	3290 ± 81	875 ± 46	4165 ± 85
	Running wheel	3561 ± 96	886 ± 68	4447 ± 106
	Environment enriched	3590 ± 168	981 ± 69	4571 ± 135

There is a significant (*P* < 0.001) effect of environment on the number of TH+ and Total (TH+ and TH−) neurons in SNc and VTA (one-way ANOVA). There is no significant (*P* > 0.05) effect of environment on TH− neurons in any nucleus and no significant (*P* > 0.05) effect of environment on TH+ and Total (TH+ and TH−) neurons in LC. SE, standard error; ANOVA, analysis of variance.

* *P* < 0.05, versus SH;

† *P* < 0.05, versus RW (Tukey multiple comparisons).

These EE-induced changes in TH+ neurons were not accompanied by coincident changes in the number of TH− SNc neurons (Table 2, but see Discussion).

### GABA_A_ receptor blockade abolishes the EE-induced increase in number of TH+ SNc neurons

**Figure 3:**
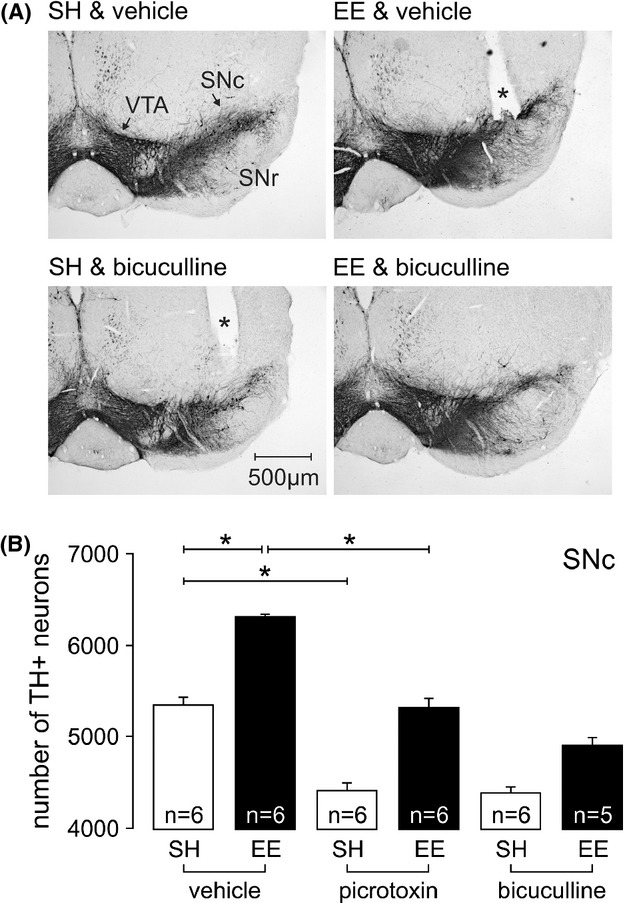
GABA_A_ receptor blockade completely abolishes the environment enrichment-induced increase in number of tyrosine hydroxylase immunoreactive (TH+) neurons in the substantia nigra pars compacta (SNc). (A) Photomicrographs through SNc showing TH immunoreactive neurons (black reaction product) in SH and EE mice receiving local infusion into SNc of vehicle or the GABA_A_ receptor antagonist 5 μmol/L bicuculline. SNr, substantia nigra pars reticulata; VTA, ventral tegmental area; *damage caused by the infusion cannula (the infusion cannula was located at a different rostrocaudal level of SNc in the other sections). (B) Mean ± SE number of TH+ SNc neurons in mice subjected to different environments (SH or EE) and different drug infusions (vehicle, 10 μmol/L picrotoxin, or 5 μmol/L bicuculline). *Significant difference (two-way ANOVA with Tukey multiple comparisons, see Table 3) between vehicle- and picrotoxin-infused mice (the same differences exist between vehicle- and bicuculline-infused mice also, but are not shown). SE, standard error; ANOVA, analysis of variance; SH, standard housed; EE, environment-enriched.

Local infusion into SNc of the GABA_A_ receptor antagonist 10 μmol/L picrotoxin or 5 μmol/L bicuculline significantly reduced the number of TH+ SNc neurons in SH mice (Fig. 3A and B; Table 3). In EE mice, infusion of either GABA_A_ antagonist completely abolished the EE-induced increase in number of TH+ SNc neurons seen in vehicle-infused EE mice (Fig. 3A and B; Table 3).

**Table 3:** Stereological estimates of the number (mean ± SE, *n* = 6/group) of tyrosine hydroxylase immunopositive (TH+) and immunonegative (TH−) neurons in the substantia nigra pars compacta (SNc) in environment-enriched adult male mice receiving local infusion into SNc of vehicle (veh.), the GABA_A_ receptor antagonist 10 μmol/L picrotoxin (Pic.), or 5 μmol/L bicuculline (Bic.)

		TH+	TH−	Total (TH+ and TH−)
SNc	Standard housed + veh.	5343 ± 78	730 ± 61	6072 ± 94
	Standard housed + Pic.	4401 ± 142*	855 ± 66	5256 ± 98*
	Standard housed + Bic.	4384 ± 62*	1119 ± 42*	5503 ± 82*
	Environment enriched + veh.	6301 ± 50	799 ± 41	7101 ± 44
	Environment enriched + Pic.	5321 ± 101*	1589 ± 45*	6911 ± 100
	Environment enriched + Bic.	4903 ± 105*	1466 ± 63*	6369 ± 101*

There are significant (*P* < 0.001) main effects of environment and of drug on the number of TH+, TH−, and Total (TH+ and TH−) neurons in SNc (two-way ANOVA). There is a significant (*P* < 0.001) environment × drug interaction effect on the number of TH− and Total (TH+ and TH−) neurons in SNc (two-way ANOVA). SE, standard error; ANOVA, analysis of variance.

* *P* < 0.05, drug versus vehicle (Tukey multiple comparisons).

EE with vehicle infusion had no effect on the number of TH− SNc neurons (Table 3), confirming the earlier result (Table 2). In contrast, infusion of either GABA_A_ receptor antagonist during EE resulted in significantly more TH− SNc neurons than EE mice receiving vehicle (Table 3). Note also that SH mice receiving bicuculline had significantly more TH− SNc neurons than SH mice receiving vehicle (Table 3).

## Discussion

### Summary

Here, we describe changes in the number of TH immunoreactive (TH+) neurons in the adult mouse midbrain (SNc and VTA) associated with environmental and behavioral manipulations. More TH+ neurons were present in males paired with females (mated males) whereas less TH+ neurons were present in females paired with males (mated females). Also, more TH+ neurons were present in males housed in an environment conducive to increased exploratory and sensorimotor activities (environment enrichment or EE). These data indicate that the number of DA neurons in the adult midbrain is not fixed, but readily increases and decreases in response to environmental stimuli and/or behavior. Furthermore, the EE-induction of more SNc TH+ neurons was abolished by blockade of midbrain GABA_A_ receptors. This indicates that environmental/behavioral influences on the number of DA neurons in the adult SNc are mediated by changes in afferent input.

### Mechanisms of changes in number of DA neurons

Our hypothesis is that changes in the number of SNc DA neurons are due to DA phenotype acquisition or loss by mature neurons located within SNc, rather than by neurogenesis or apoptosis. This is based on the following: (1) local infusions of ion-channel agonists and antagonists result in equal but opposite changes in the number of TH+ and TH− SNc neurons, that is, there is no net change in number of SNc neurons (TH+ and TH− combined) (Aumann et al. 2008, 2011); (2) the magnitude and rate of recruitment of additional TH+ neurons (typically ∼500 more TH+ cells within 2 weeks of drug infusion (Aumann et al. 2011, 2008) or environmental/behavioral manipulation (present study) seems too high to be due to DA neurogenesis, which occurs either not at all (Lie et al. 2002; Zhao et al. 2003; Cooper and Isacson 2004; Frielingsdorf et al. 2004; Chen et al. 2005; Yoshimi et al. 2005; Shan et al. 2006; Aponso et al. 2008; Peng et al. 2008; Hermann et al. 2009) or at very low rates (Zhao et al. 2003; Shan et al. 2006) in adult mouse SNc; and (3) evidence indicates similar changes in hypothalamus are due to DA neurotransmitter “switching,” not neurogenesis or apoptosis (Dulcis et al. 2013). Blockade of GABA_A_ receptors in EE mice (Table 3) supports this hypothesis by providing instances of reciprocal changes in the number of TH+ and TH− SNc neurons. However, while the number of TH+ SNc neurons changed in mated and environment-enriched mice without drug infusion, the number of TH− SNc neurons did not (Tables 1, 2). Nevertheless this could be due to an artifact of the stereological method rather than evidence against DA neurotransmitter switching. We noted in the present study that many “new” TH+ neurons lay just outside the perimeter of SNc following behavioral manipulation. Therefore, in these mice the borders of SNc (defined by the stereologist as the area circumscribing TH+ SNc cells) would expand compared to control mice (e.g., SH). Intermingled among these “new” TH+ neurons are TH− neurons which would otherwise be considered outside SNc and therefore not be counted. These additional TH− neurons would offset any decrease in TH− neurons brought about by acquisition of the DA phenotype. In the case of DA phenotype loss (e.g., mated females in Fig. 1A), the opposite might occur (i.e., the borders of SNc contract and any increase in TH− neurons due to DA phenotype loss would be offset by exclusion of perimeter TH− neurons from the counts). Further, we have previously shown that reciprocal changes in TH+ and TH− SNc neurons occur in almost every instance of drug infusion only (i.e., without concurrent EE) (e.g., SH + bicuculline in the present study [Table 3] or [Aumann et al. 2011, 2008]), indicating that SNc borders remain stable in this context. Taken together, these data indicate that the environment or behavior has access to a broader pool of “switchable” SNc neurons than drug infusion, and most of these are located on the perimeter of SNc. When we subject mice to environmental manipulations alone (e.g., mating or EE in the present study), we do not see reciprocal changes in the number of TH+ and TH− SNc neurons for the reasons just discussed. However, when we infuse drugs alone (e.g., SH + bicuculline in the present study [Table 3] or [Aumann et al. 2008, 2011]), or combine environmental manipulations with drug infusions (e.g., environment + picrotoxin or EE + bicuculline in the present study [Table 3]), reciprocal changes become apparent because the borders of SNc remain unchanged in perturbed mice with respect to their controls.

Whether or not the present findings reflect DA phenotype switching by a population of midbrain neurons remains to be determined. They are unlikely to be due to neurogenesis (as discussed above) but they may reflect levels of TH protein rising above or falling below detection threshold. Nevertheless our data show, at the very least, that the environment or behavior produces significant and substantial changes in TH protein in adult mouse midbrain neurons. This ought to cause significant and substantial changes in DA synthesis, DA signaling, brain function, and behavior.

The abolition of environment enriched increases in TH+ SNc (and VTA) neurons by GABA_A_ receptor blockade gives further clues about mechanisms. At least 70% of afferents to SNc are GABAergic and the vast majority of these arise from the striatum, globus pallidus externa (GPe), and substantia nigra pars reticulata (SNr) (Tepper and Lee 2007). Also, GABA_A_ and not GABA_B_ receptors are the predominant, perhaps exclusive mediators of inhibition in SNc neurons evoked from these locations (Tepper and Lee 2007). Thus, if afferent pathways mediate these changes in TH+ cell number, they most likely relay through striatum, GPe, and/or SNr. Previously we identified striatal D2 DA receptors as much more potent in regulating the number of SNc TH+ neurons than striatal D1 DA receptors (D2 and D1 receptors in SNc were also ineffective in this regard) (Aumann et al. 2011). Therefore the indirect pathway from the striatum to GPe to SNr to SNc is emerging as a strong candidate mediating environment and/or behavioral influences over the number of SNc TH+ neurons.

In addition, in catecholaminergic neurons generally, an increase in neuronal activity is linked with an increase in TH expression (Zigmond et al. 1980; Baker et al. 1983; Black et al. 1985; Biguet et al. 1989; Schalling et al. 1989; Liaw et al. 1992). We too have reported evidence for this in isolated midbrain preparations (Aumann et al. 2011). Therefore, it is counterintuitive that local blockade of GABA_A_ receptors (which ought to increase neuronal activity and TH expression) results in fewer TH+ SNc neurons (e.g., Fig. 3A and B; Table 3). However, the effects of GABAergic synapses on the activity of SNc neurons is complex. Although local blockade of GABA_A_ receptors causes SNc neurons to shift from tonic toward burst firing, there is very little effect on their overall firing rate in anesthetized rats (Tepper and Lee 2007). Also, the effects of endogenous GABA signaling, induced by electrical stimulation of striatum, GPe or SNr, is often complex and depends on the type and intensity of stimulation (Tepper and Lee 2007). Furthermore, there are multiple GABAergic relays onto SNc neurons that are in close proximity in midbrain. For example, SNr neurons receive GABAergic synapses and also provide GABAergic synapses to SNc. SNr neurons are also more sensitive to GABA-mediated inhibition than SNc neurons due to differential expression of chloride transporters and different chloride reversal potentials (Tepper and Lee 2007). Thus, our GABA_A_ antagonist infusions may be preferentially exciting SNr neurons leading to inhibition of SNc neurons. Spatiotemporal calcium transients linked to neuronal activity, and their downstream signaling pathways, are also likely to provide additional layers of complexity over putative activity-dependent regulation of TH gene expression (Aumann and Horne 2012).

### Types of behavior regulating the number of DA neurons

The precise environmental or behavioral factors influencing the number of midbrain DA neurons are not known. In the mating experiment, any or all of odors, pheromones, hormones, moods, stressors, social interactions, cognitive, and motor behaviors could be involved. For example, sex steroids are involved because female rodents have more midbrain DA neurons than males (Walker et al. 2012), confirmed in the present study (Fig. 1, control female vs. control male), and manipulating estrogen or testosterone signaling changes the number of midbrain neurons (Johnson et al. 2010a,b). Also, females have greater striatal DA release and uptake consistent with a higher density of DA terminals (Walker et al. 2000), and there are gender differences in striatal DA function (Becker 1999). With regard to SNc versus VTA, the general consensus is SNc is more involved with motor behavior, VTA with cognitive aspects of behavior. The similarities between the two nuclei (i.e., DA delivery into different areas of striatum) suggest they perform a similar function on these different aspects of behavior, and that function appears to be learning. Using the example of SNc, the process of making movements stereotyped is a fundamental role of corticostriatal circuits. The biological substrate for “laying down” these stereotyped movements includes DA delivered into the striatum by SNc neurons, where it regulates corticostriatal synaptic plasticity (Calabresi et al. 2007). SNc DA neurons burst in response to presentation of rewards and this burst shifts back in time to any cue that is associated by the animal to forthcoming rewards (i.e., to just before movement to retrieve a reward) (Schultz and Romo 1990; Schultz et al. 1997). SNc DA neurons also burst strongly during early trials when movement errors are frequent and success at retrieving reward unpredictable, but progressively less as performance is consolidated (Hollerman and Schultz 1998). This is consistent with a requirement for more DA during learning of movements that provide some advantage (e.g., food), and this may be a time when DA phenotype recruitment occurs also.

It was in this context that male mice were placed into an environment in which motor skill learning was a major variable and the possible influences of smells, pheromones, hormones, moods, stressors, and social interactions were better controlled, that is, environment enrichment or EE. EE resulted in a larger (on average) increase in number of TH+ SNc (and VTA) neurons than did mating (compare Figs. 1, 2; Tables 1, 2). This may reflect a greater motor component to the salient or learned behaviors required in EE. In this context it is relevant that the RW alone produced only a nonsignificant trend to more SNc DA neurons than mice without RWs (compare RW to SH mice in Fig. 2A; Table 2). This further argues that it is not simply the presence of “motor” activity that is necessary for the increase in TH+ SNc neurons but that factors such as novelty and salience are important. During EE, mice are not only more active but there is more variety and novelty (in the form of new toys for 1 h/day, 5 days/week [“super enrichment” – see Methods]). Clearly we cannot differentiate these factors in the present data, however, in light of the putative role of SNc DA in motor learning discussed above, it will be interesting to determine in future experiments if novel motor behavior is the important variable.

### Consequences of changes in number of DA neurons

Presumably changes in the number of DA neurons in SNc will lead to changes in DA signaling in the striatum. This would be expected to regulate DA-dependent corticostriatal plasticity to reorganize circuitry governing motor performance. This raises the question of why recruit more DA neurons when increased DA could equally be achieved by increasing DA synthesis in existing DA neurons? One plausible reason is to mitigate increased toxicity that would accompany increased DA synthesis in DA neurons. DA and DA metabolites are toxic to neurons (Stokes et al. 1999) and mechanisms to protect against this toxicity are elevated in SNc DA neurons (Calabrese et al. 2002). However, presumably there is an upper limit to this protection which, if exceeded (e.g., by increasing DA synthesis too much), will cause cell death. Recruitment of new DA neurons would be a way to increase the amount of brain DA without exceeding this toxic threshold.

## Conclusions

The data reported here and the ensuing discussion support the notion that there is a substantial population of neurons in the adult midbrain, including SNc, that switch between the DAergic and non-DAergic phenotypes according to afferent input relaying information about the environment or the behavioral state of the animal. To our knowledge this is the first reported evidence that environmental stimuli and/or behaviors change the number of DA neurons in the adult midbrain of any species. This may be an important novel form of brain plasticity mediating adaptive behavior. It may also be a mechanism underlying the reported benefits of cognitive behavioral therapies (CBTs) on diseases and disorders (or symptoms thereof) associated with midbrain DA imbalances (e.g., Parkinson's disease, Tourette's syndrome, obsessive compulsive disorder, attention deficit hyperactivity disorder, depression, schizophrenia, and drug addiction). Finally, identifying midbrain cells that can switch phenotype may provide valuable information for cell-replacement strategies to treat these disorders. It may be sufficient to replace these “switchable” cells and let the environment or behavior undertake the difficult task of inducing the DA phenotype in situ.
